# Supplementary material for: Transiently delocalised hybrid quantum states are gateways for efficient exciton dissociation at organic donor-acceptor interfaces
Source: Nat Commun. 2025 Dec 22;16:11560. doi: 10.1038/s41467-025-67722-4 (PMC12750018; doi:10.1038/s41467-025-67722-4)
Supplement: Supplementary file 1 — Supplementary Information [file 41467_2025_67722_MOESM1_ESM.pdf]

Supplementary Information: Transiently Delocalised Hybrid Quantum States are Gateways for Efficient Exciton Dissociation at Organic Donor-Acceptor Interfaces

Filip Ivanović[1], Samuele Giannini[2], Wei-Tao Peng[3], Jochen Blumberger[1*]

[1*]Department of Physics and Astronomy and Thomas Young Centre, University College London, Gower Street, London, WC1E 6BT, United Kingdom.

[2]Department of Chemistry and Industrial Chemistry, University of Pisa, Via Giuseppe Moruzzi, Pisa, 56124, Italy.

[3]Department of Chemistry, Tunghai University, Taiwan Boulevard, Taichung City, Section 4, Xitun District, Taiwan.

*Corresponding author(s). E-mail(s): j.blumberger@ucl.ac.uk; Contributing authors: ucanfiv@ucl.ac.uk; samuele.giannini@cnr.it; pengwt@go.thu.edu.tw;

Supplementary Figure 1: Selection of Initial States

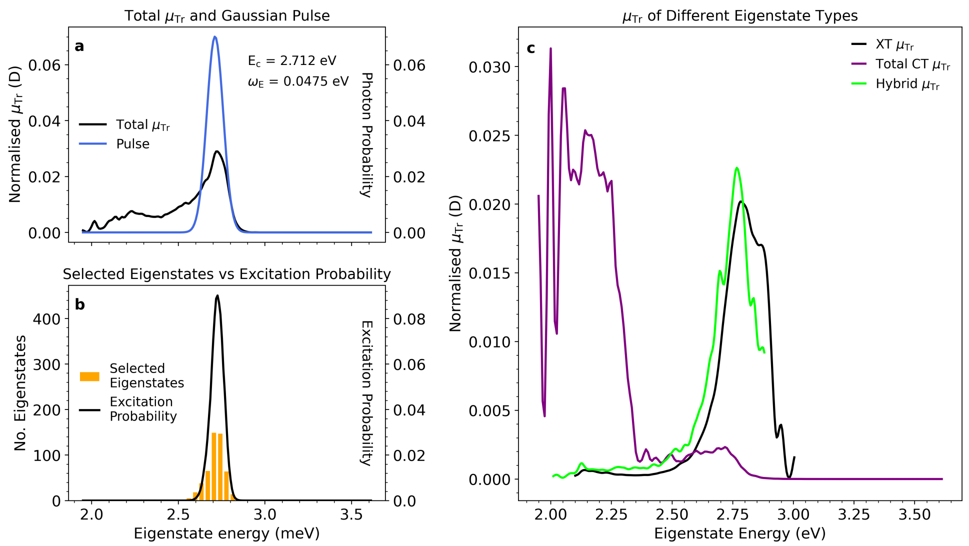

Supplementary Fig. 1 Plots of the exciting Gaussian pulse, transition dipole moment, and total excitation probability. The black curve in (**a**) plots the binned eigenstate energy (relative to the neutral ground state) against μ_{Tr} of each bin, using an ensemble of 420,000 eigenstates obtained from diagonalising the electronic Hamiltonians of an ensemble of 1000 nuclear configurations, which were sampled from a 1 ns NVE run in the neutral ground state. The blue curve is the plot of the exciting (10 fs) Gaussian pulse in the energy domain, where the central wavelength has been selected to maximally overlap with the peak of μ_{Tr}. The annotations E_{c} and ω_{E} denote the central pulse energy and standard deviation, respectively. The orange histogram in panel (**b**) displays the distribution of 500 selected eigenstate energies for simulation (1), sampled from the aforementioned ensemble of eigenstates. The black curve shows the excitation probability of the total eigenstate ensemble as a function of its energy, when the ensemble has been collected into 200 energy bins, with each bin having an average excitation probability. Each curve in panel (**c**) shows the transition dipole moments obtained from only considering the XT (black), hybrid XT-CT (lime), and CT (purple) eigenstates.

Supplementary Figure 2: Electronic Populations

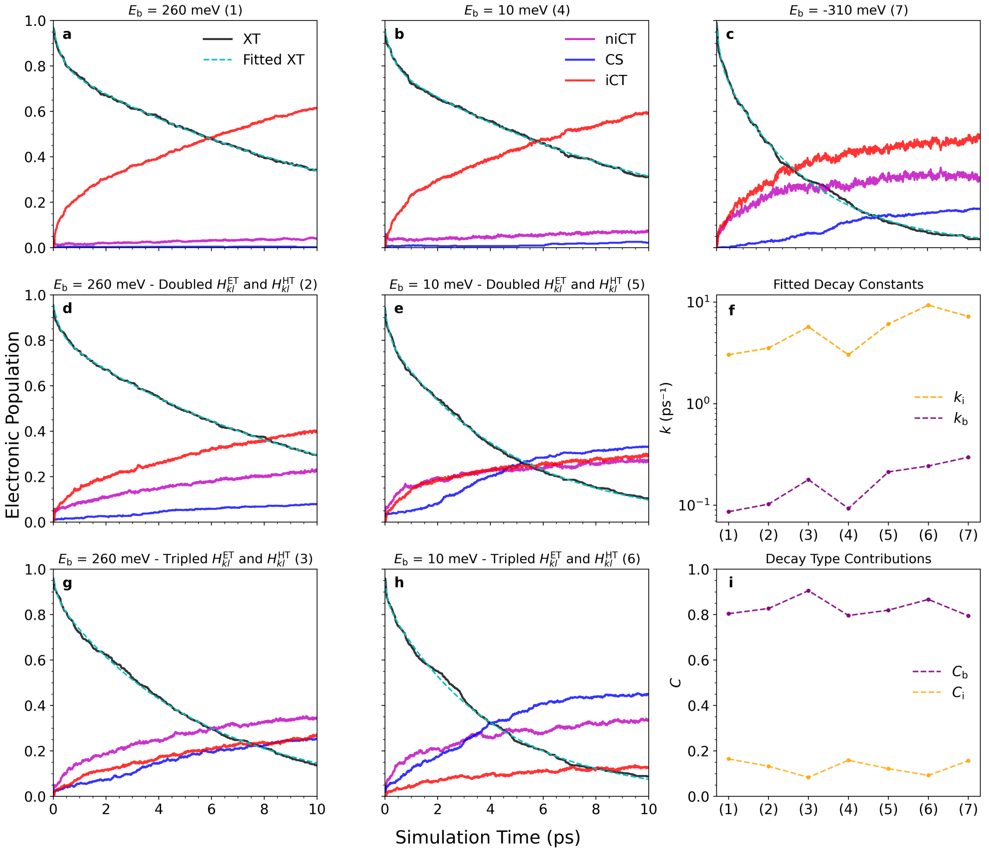

Supplementary Fig. 2 Kinetics of exciton dissociation and charge generation for all simulations. The electronic populations are given by Eqs. 17-20 in the main text. P_{XT} plots are overlaid with exciton decay profiles predicted from the least-squares fitting of a biexponential decay function to the X-SH data (Eq. 1, main text). The simulation label (Table 2, main text) of each panel is included in its title. Panels (**a**), (**b**), and (**c**) display the populations when the electronic coupling is unmodified and $E_{\mathrm{b}} = 260$, 10, and -310 meV, respectively. Panels (**d**) and (**f**) correspond to $E_{\mathrm{b}} = 260$ meV with progressively stronger electronic couplings between CT states. Panels (**e**) and (**g**) additionally refer to $E_{\mathrm{b}} = 10$ meV with increasing couplings. Panel (**f**) plots the fitted decay constants associated with the dissociation of bulk and interfacial excitons obtained from least-squares fitting; the numbers on the x-axis refer to the simulation labels presented in Table 2 of the main text, which are also included in the titles of all population plots. Panel (**i**) displays the relative proportions of excitons initialised in the bulk region of the acceptor phase or close to the interface. The values of C were constrained during optimisation such that their sum is always equal to P_{XT} at $t = 0$.

Supplementary Figure 3: Least-squares Fitting

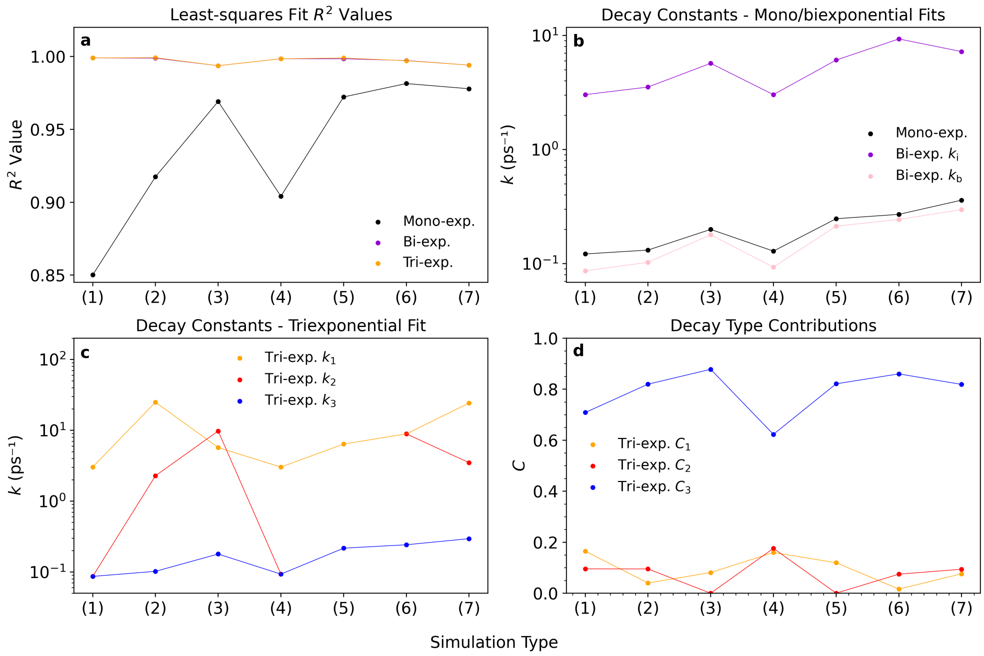

Supplementary Fig. 3 Validation of the least-squares fitting of exciton populations. (**a**) Plot of R^2 values obtained from the least-squares fit of exponential decay functions with 1 (black), 2 (purple), and 3 (orange) decay terms, to P_{XT} of each simulation in the main text. (**b**) Plot of the decay constants obtained from the least-squares fit of the 1-term and 2-term exponential decay functions. The decay constants obtained from the single exponential fit are shown in black, whilst the bulk decay constants (k_{b}) obtained from the bi-exponential decay function are shown in pink, and the fast interfacial decay constants (k_{i}) are shown in purple. (**c**) Plot of the decay constants obtained from the least-squares fit of an exponential decay function with 3 terms. The first two decay constants, k_1 and k_2, are plotted in orange and red, respectively. The value of k_2 is not displayed for (5) and is instead included here: $k_2 = -0.335$ ps^{-1}, with its corresponding contribution to exciton dissociation being negligible. (**d**) Plot of the contributions of each term in the tri-exponential decay fit. The respective contributions of the k_1, k_2, and k_3 decay terms are plotted in orange, red, and green, respectively.

Supplementary Figure 4: Exciton Dissociation Distance

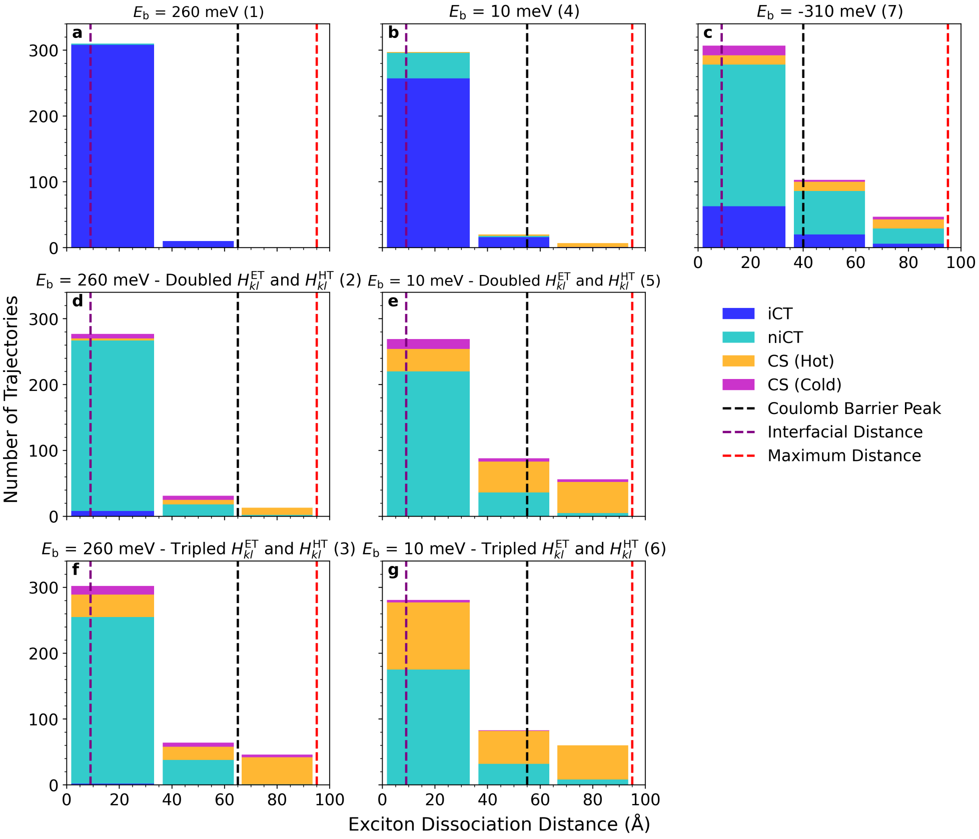

Supplementary Fig. 4 Distributions of exciton distances at dissociation. All panels plot the exciton distance from the aT6:PDI interface immediately preceding exciton dissociation, and the fraction of excitons relaxing to iCT, niCT or CS states following dissociation. Panels (**a**), (**b**), and (**c**) display such distributions when the electronic coupling between CT states is unmodified and E_b = 260, 10, and −310 meV, respectively. Panels (**d**) and (**f**) correspond to E_b = 260 meV with progressively stronger electronic couplings between CT states. Panels (**e**) and (**g**) refer to E_b = 10 meV with increased CT couplings as well. Different coloured sections within the bar of a given distance bin denote the proportion of excitons in this bin that have relaxed into a certain electronic CT state (iCT, niCT, CS) after 10 ps. The dotted purple and red vertical lines refer to the interfacial and boundary PDI distances, respectively. The black dotted line denotes the electron-hole distance of the Coulomb barrier peak for a given E_b.

Supplementary Figure 5: Exciton Relaxation Pathways

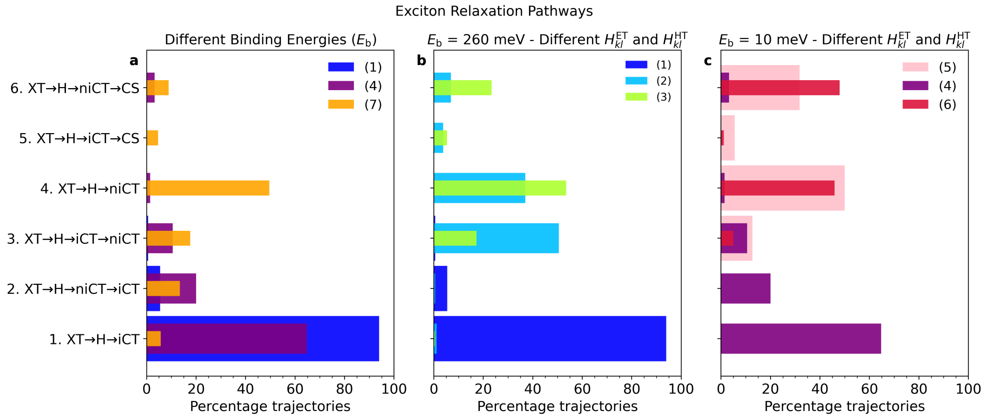

Supplementary Fig. 5 Plots of the percentage of dissociated excitons that relax via 6 general pathways in X-SH. The effect on the pathways taken due to a weaker electron-hole Coulomb attraction (hence smaller E_b) is shown in (**a**). Panels (**b**) and (**c**) display this effect for increasing the electronic coupling between CT states, when $E_\mathrm{b} = 260$ meV and $E_\mathrm{b} = 10$ meV, respectively. The pathways denote the following: 1. Relaxation of exciton directly to iCT. 2. Dissociation of exciton to niCT, which then relaxes to iCT. 3. Exciton dissociates into iCT, then carriers reach the niCT state. 4. Exciton directly dissociates into niCT. 5. CS state reached after exciton directly dissociates into iCT which then surmounts the coulomb barrier - charge generation via cold exciton dissociation. 6. Exciton dissociates into niCT state which transitions to CS without relaxing to iCT - charge generation via hot exciton dissociation.

Supplementary Figure 6: Hybrid-state IPR vs Electron-hole Distance

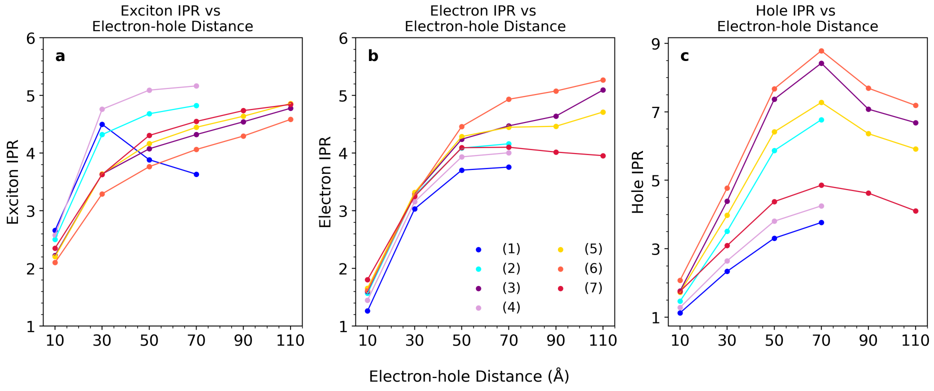

Supplementary Fig. 6 Hybrid XT-CT state IPR vs the electron-hole distance of its CT component. (**a**) Plot of exciton IPR of hybrid XT-CT eigenstates as a function of electron-hole distance, calculated by sorting an ensemble of sampled hybrid eigenstates into 2nm-wide bins, and calculating the mean exciton IPR of the hybrid XT-CT states within each bin. (**b**) Plot of hybrid state electron IPR as a function of electron-hole distance, using the same procedure as that for (**a**). (**c**) Plot of hybrid state hole IPR as a function of electron-hole distance.

Supplementary Figure 7: Averaged Hybrid-state Properties

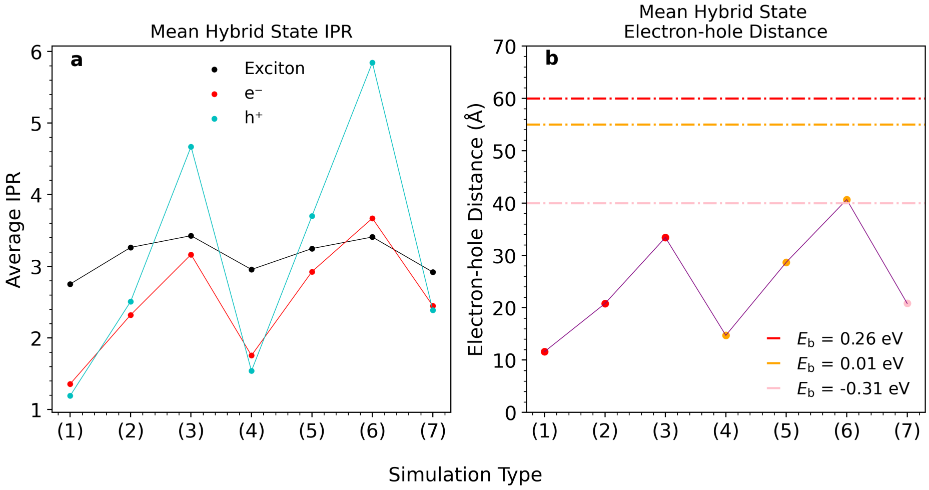

Supplementary Fig. 7 Mean particle IPR of all sampled hybrid XT-CT states from simulations (1) - (7). (**a**) Mean exciton, electron, and hole IPR, calculated from an ensemble of sampled hybridised eigenstates during the dynamics of simulations (1) - (7). (**b**) Mean expectation values of the hybrid states' electron-hole distance, calculated for simulations (1) - (7). The dash-dotted horizontal lines correspond to the electron-hole distances of the coulomb barrier peaks for exciton binding energies of 260 meV (red), 10 meV (orange), and −310 meV (pink). This shows that the hybrid states' CT-state components become energetically closer to the coulomb barrier peak as the electron-hole distance increases, which makes the hot pathway more thermally accessible. The coloured dots on the purple line denote the values of E_b of each simulation accordingly.

Supplementary Figure 8: Hybrid-state Exciton vs Electron Distance

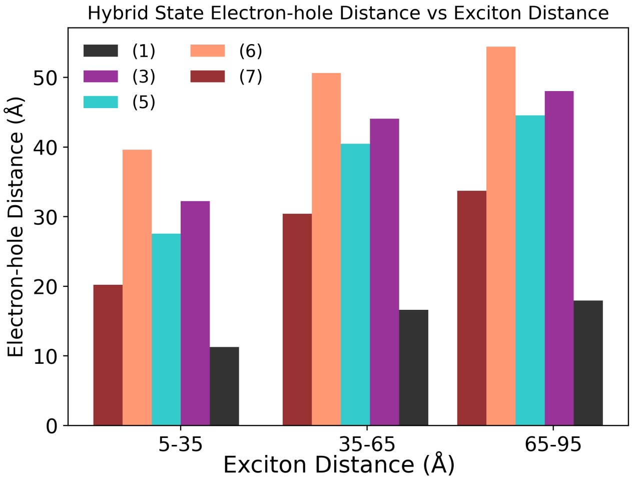

Supplementary Fig. 8 Hybrid XT-CT state electron-hole distance vs exciton distance from the interface. This was calculated by binning a ensemble of hybridised eigenstates, sampled during the dynamics of simulations (3) and (5) - (7), by their expectation values of the exciton distance, and then calculating the mean electron-hole distance of the hybrid states within each exciton distance bin.

Supplementary Figure 9: Non-adiabatic Coupling Analysis

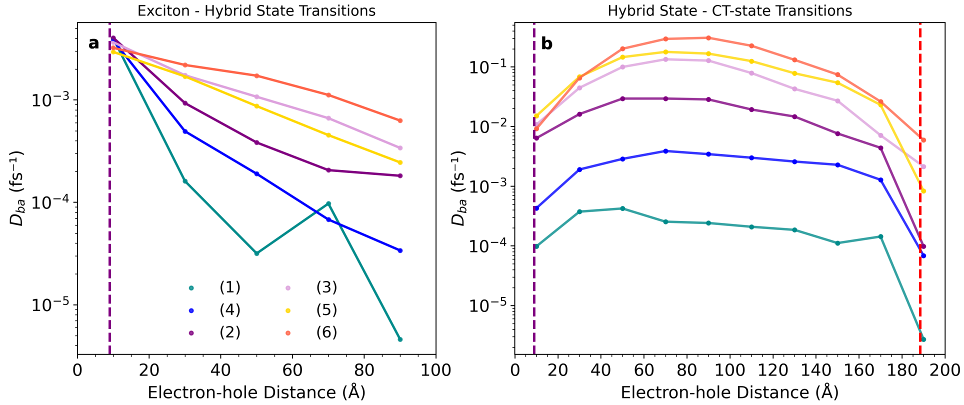

Supplementary Fig. 9 Sums of Boltzmann-weighted NACEs between different types of electronic states. The NACEs for a transition between two eigenstate types are sampled from all timesteps for each X-SH simulation, and weighted according to the potential energy difference between the active and future eigenstates. (**a**) Plot of weighted NACE sums concerning transitions between XT and hybrid XT-CT states, where the total ensemble of sampled hybrid states has been separated into 5 bins, according to the expectation value of the electron-hole distance in each hybrid state. The x-value of each point corresponds to the mid-point of a distance bin. The vertical purple line denotes the electron-hole distance of the quasi-diabatic iCT state. (**b**) Weighted NACE sums of transitions between hybrid XT-CT states and pure CT states, where the CT states are now resolved by their expectation value for the electron-hole distance. The red dotted line denotes the maximum possible electron-hole distance in the electronically active region.

Supplementary Figure 10: Convergence of Exciton Dissociation Distance

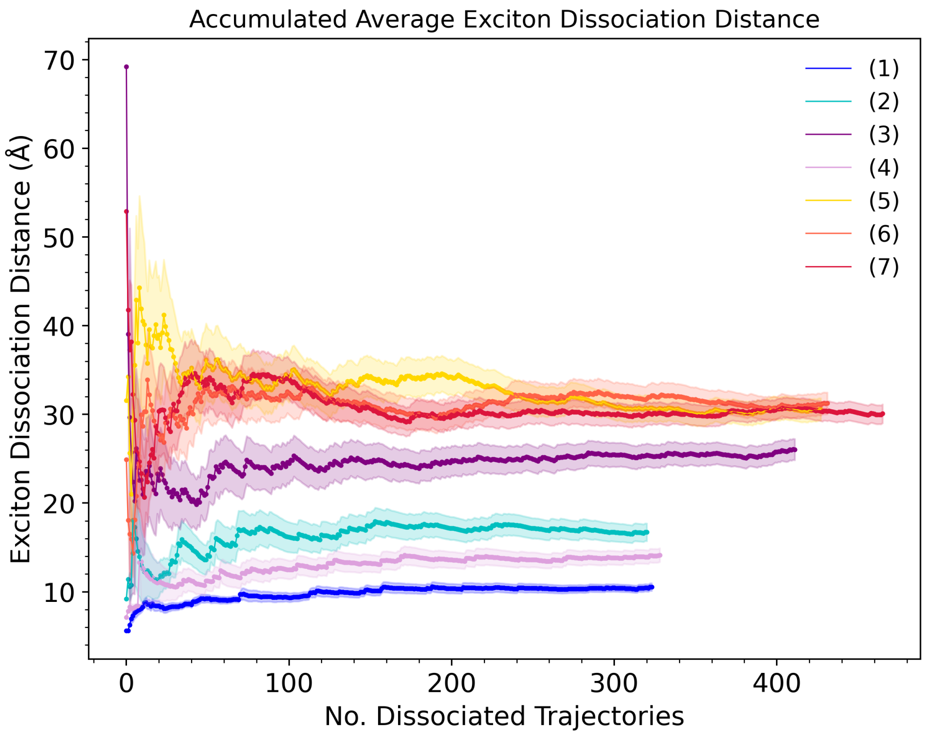

Supplementary Fig. 10 Accumulated averages of exciton dissociation distance with respect to the interface. The accumulated averages are shown by the lines and dots, whilst the error bars, given by the standard error at each x-value, are plotted as shaded regions. The mean dissociation distance is plotted against the number of dissociated trajectories in each simulation.

Supplementary Figure 11: Convergence of Exciton Dissociation Mechanism

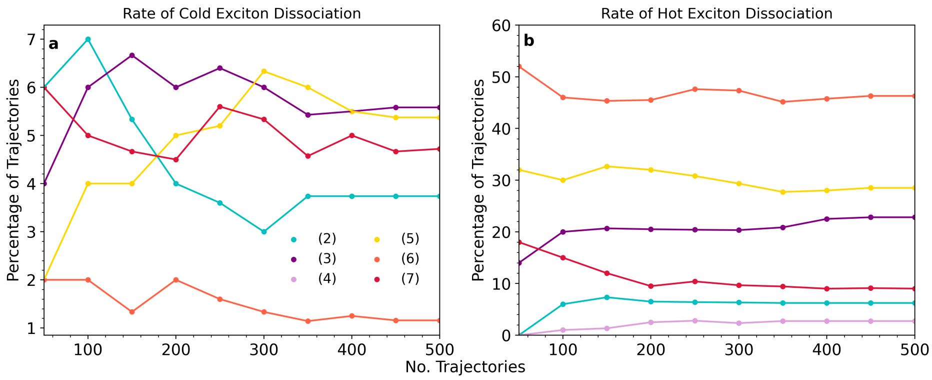

Supplementary Fig. 11 Convergence of pathways of charge generation with respect to the number of trajectories sampled. (**a**) Plot of percentage contribution of the cold exciton dissociation pathway, as a function of trajectories in X-SH, for simulations (2) - (7). Convergence has been achieved for all simulations after 400 trajectories. (**b**) Plot of percentage contribution of the hot exciton dissociation pathway as a function of X-SH trajectories, with convergence being achieved when more than 300 (10 ps) trajectories have been sampled. The percentages have been calculated with respect to the total number of dissociated trajectories; trajectories where the exciton has not yet dissociated have been neglected when sampling the decay pathways of the trajectories.

Supplementary Figure 12: Energy Conservation

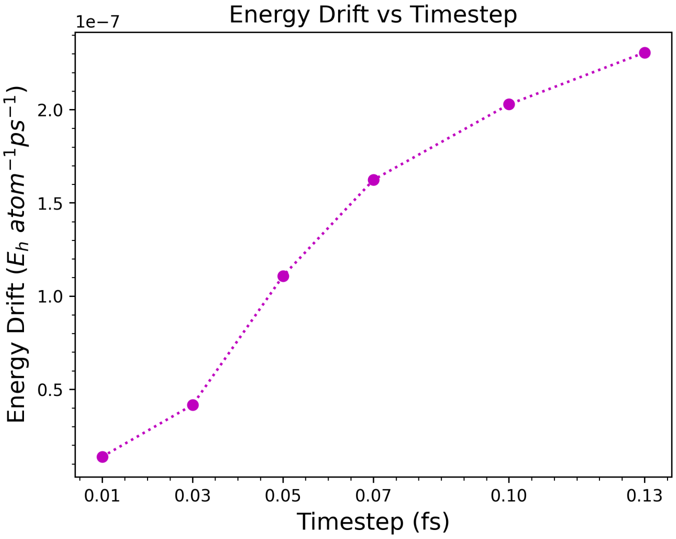

Supplementary Fig. 12 Energy drift as a function of nuclear timestep. The energy drift has been averaged over 100 X-SH trajectories for a two-dimensional reference system with 930 quasi-diabatic states. Each trajectory lasted 300 fs, and the simulation was repeated for 6 different nuclear timesteps, ranging from $0.01 - 0.13$ fs. All nuclear timesteps provided energy drifts that were comparable to that observed in previous work.

Supplementary Figure 13: Convergence of Exciton Dissociation with Nuclear Timestep

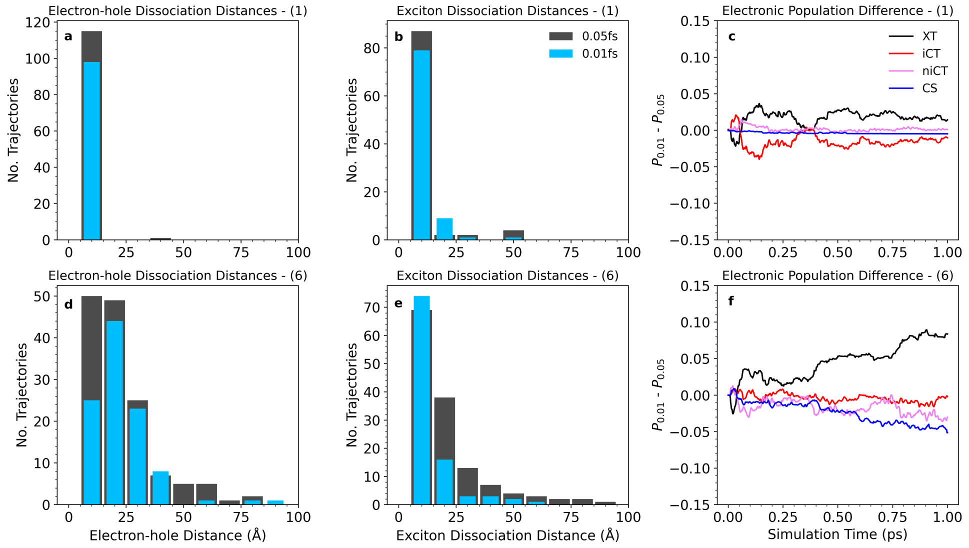

Supplementary Fig. 13 Convergence of key observables with nuclear timestep for simulations (1) and (6). Panels (**a**) and (**d**) display distributions of electron-hole distances immediately following exciton dissociation, for all trajectories that have exhibited exciton dissociation within 1 ps. Exciton dissociation in a single trajectory is taken as P_{XT} decreasing below 0.05, and not increasing above this cutoff for the next 0.5 ps, as outlined in Supplementary Discussion 6. Panels (**b**) and (**e**) display distributions of exciton distances, with respect to the heterojunction interface (Eq. 13), that immediately precede exciton dissociation. Panels (**c**) and (**f**) plot the signed differences in the electronic populations of different types of quasi-diabatic states, as defined by Eqs. 17-20 in the main text, due to the reduction of the nuclear timestep from 0.05 to 0.01 fs.

Supplementary Figure 14: Validation of Electronic Coupling Calculation

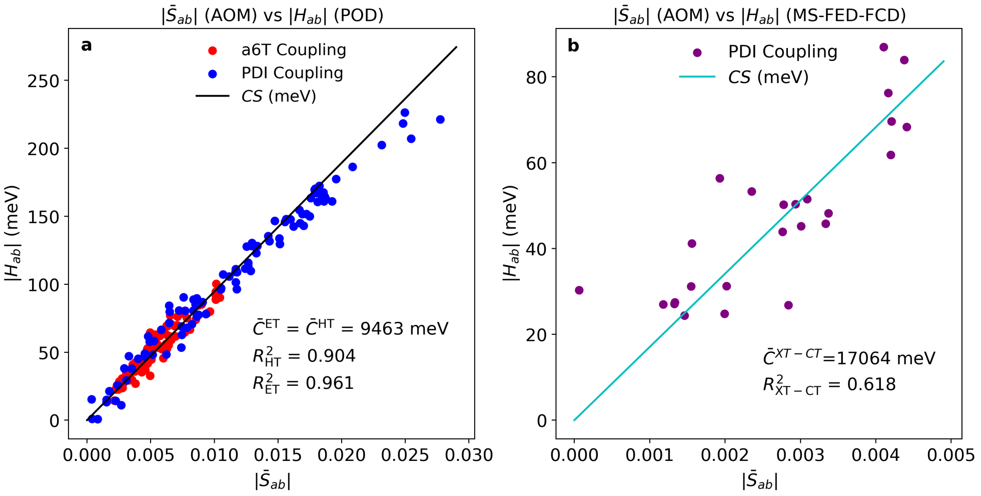

Supplementary Fig. 14 Validation of AOM electronic coupling against DFT. Panel (**a**) plots electronic couplings via hole transfer between adjacent a6T molecules or electron transfer between PDI molecules, calculated using DFT (and POD), against the relevant orbital overlaps calculated using AOM. Each point corresponds to a single dimer geometry sampled from a 1 ps X-SH run at 300 K. Panel (**b**) plots the electronic coupling between an interfacial PDI exciton and a quasi-diabatic iCT state for an interfacial a6T:PDI dimer, obtained from TDDFT and subsequent MS-FED-FCD diabatisation, against the HOMO-HOMO overlaps calculated by AOM for the same dimer geometries.

Supplementary Figure 15: 6T:PDI Interface with Defect

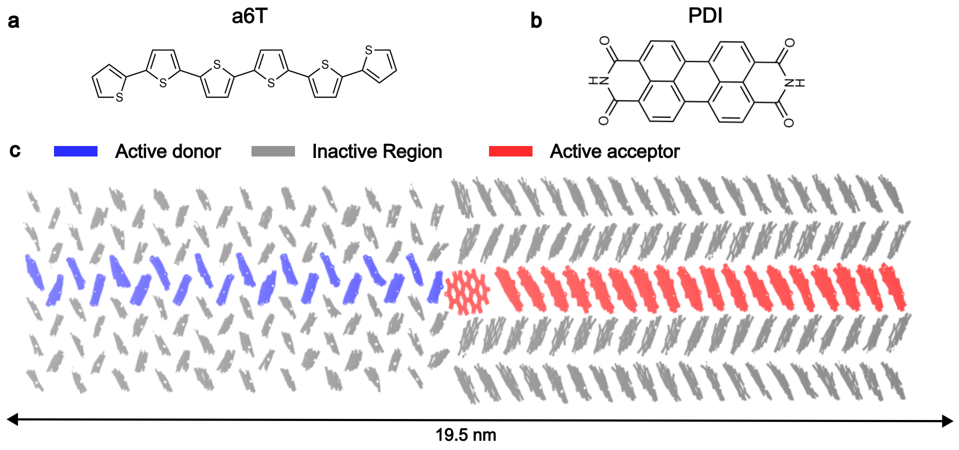

Supplementary Fig. 15 Pictorial representation of the a6T:PDI heterojunction with an interfacial defect in the PDI phase. Panels (**a**) and (**b**) depict the skeletal formulae of the donor and acceptor molecules, respectively. Panel (**c**) shows a snapshot of the heterojunction structure.

Supplementary Discussion 1: Chemical Master Equation Approach

To determine how the populations of the different electronic states would vary if the system was restricted to site-to-site hopping, we set up a chemical Master equation model for the same electronic state space as X-SH, where population transfer was restricted to localised states with charges or excitations centred on adjacent molecular sites. The rate constants for all electron transfer or excitation energy transfer steps were calculated with semiclassical Marcus theory[1]. The time-dependent populations of all states were obtained analytically using the procedure outlined in previous work[2]. Reorganisation energies associated with given a type of transfer were set to those used in X-SH (Table 1, main text). Driving forces between different CT states were set to their differences in the Coulomb electron-hole interaction and the electric field. The energy difference between the interfacial XT and iCT states was calculated by first sampling the potential energies of eigenstates in simulation (1) where XT or CT amplitudes were $> 90\%$ localised on the interfacial PDI or the interfacial a6T:PDI pair, respectively. The mean values of these distributions were subtracted to estimate the energy difference between the localised XT and iCT states. The effective nuclear frequency of all transfer reactions was set equal to a typical value for organic molecules[3] 1539 cm^{-1}. The electronic or excitonic coupling for each reaction step was taken to be the corresponding root-mean-square coupling obtained from the X-SH simulation in the respective parameter regime. The activation free energies were estimated by first plotting the free energy surfaces of the ground and excited adiabatic states, resulting from the coupling between two localised diabatic states, in the vicinity of the energy of the transition state (when the diabatic energy gap ΔE is zero) [4]. In the normal Marcus regime we take the activation free energy in a given direction to be the difference in free energy at the corresponding minimum and the transition state (at $\Delta E = 0$) on the ground state adiabatic free energy surface. In the inverted Marcus regime, the activation free energy is taken to be the difference in free energy at the corresponding minimum and the transition state on the excited state adiabatic free energy surface. We note that in the simulations where electronic couplings are doubled (simulations 2 and 5 in Table 2 main text), the activation energy for ET in the PDI acceptor phase disappears because $\langle |H_{kl}^{\text{ET}}| \rangle > \frac{1}{2}\lambda^{\text{ET}}$. For the sake of comparing X-SH dynamics to the chemical master equation approach, we take in these instances the activation free energy to be zero in the downhill direction or equal to the driving force in the uphill direction, respectively.

Supplementary Discussion 2: Preparation of Initial States in X-SH

Theory. The initial active eigenstate in an X-SH trajectory, $\psi_a(\mathbf{R}(t=0))$, was chosen randomly with a probability $P(a, \mathbf{R})$ proportional to the probability of sampling a nuclear configuration $\mathbf{R}$ on the ground state potential energy surface of the a6T:PDI interface, $P(\mathbf{R})$, and the probability for vertical electronic excitation from the ground

to the excited eigenstate a in configuration $\mathbf{R}$ when the system interacts with a time-dependent radiation pulse, $P_{\mathbf{R}}(a)$,

$$P(a, \mathbf{R}) \propto P(\mathbf{R}) P_{\mathbf{R}}(a) \tag{1}$$

The initial electronic wavefunction in X-SH, $\Psi(0)$, is then set equal to the initial active adiabatic electronic state, $\Psi(0) = \psi_a(0)$. The probability $P(\mathbf{R})$ is obtained from MD simulation of the a6T:PDI heterojunction in the electronic ground state (see below for details of the simulation.) To estimate the probability for transition to an excited eigenstate i, $P_{\mathbf{R}}(i)$, we write the electronic wavefunction in the eigenstate basis of the electronic Hamiltonian $\hat{H}(\mathbf{R})$ as:

$$\Psi(t) = \sum_{j=1}^{M} c_j(t) \left|\psi_j\right\rangle + c_0(t) \left|\psi_0\right\rangle \tag{2}$$

where $c_j(t)$ and $c_0(t)$ are the expansion coefficients of a given excited eigenstate, j, and the ground state, respectively. For ease of notation the dependence of the eigenstates on $\mathbf{R}$ is dropped. We assume that the interaction between the electric field of the pulse and the dipoles of the PDI molecules results in a perturbative electronic coupling between the ground state and an excited state. We also assume that the electronic transition between the states occurs vertically. We approximate the time-dependent field of the pulse to be a packet of classical light waves with associated angular frequencies, ω, each having a certain contribution to the overall wavepacket [5]. The electric field of the pulse is then approximated by an integral over the distribution of angular frequencies, and the distribution itself is assumed to be Gaussian around a central frequency:

$$E(t) = \mathbf{e}\frac{1}{2}\int d\omega E_0(\omega)[e^{i\omega t} + e^{-i\omega t}] \tag{3a}$$

$$E_0(\omega) = E_{\max}(2\pi\sigma_\omega^2)^{-1/2} e^{\frac{(\omega-\omega_c)^2}{2\sigma_\omega^2}} \tag{3b}$$

where the polarisation vector is given by $\mathbf{e}$, the frequency distribution is given by $E_0(\omega)$, and the sum of the $\exp(\pm i\omega t)$ terms corresponds to the wave equation. We take the assumption that all waves comprising the pulse are parallel in $\mathbf{e}$, which is often the case in laser pulses [5]. The maximum electric field of the pulse is given as $E_{\max}$. The central frequency and standard deviation of the Gaussian distribution are ω_c and σ_ω, respectively. The pre-exponential factor of the $E_0(\omega)$ term in Eq. 3 is the normalisation constant of the Gaussian frequency distribution. By substituting Eq. 3b into Eq. 3a and evaluating the Fourier transform, one obtains the Gaussian pulse in the time domain:

$$E(t) = \frac{\mathbf{e}E_{\max}}{2} e^{\frac{-t^2}{2\sigma_t^2}} [e^{i\omega_c t} + e^{-i\omega_c t}] \tag{4}$$

where σ_t is the standard deviation of the Gaussian distribution in the time domain. The pulse couples the ground and excited eigenstates through the interactions of its

electric field with the total molecular dipole moment of the system, given by the vector operator $\hat{\boldsymbol{\mu}}_{\mathrm{tot}}$. We take this as a time-dependent perturbation that can be written as:

$$V_{i\leftarrow 0}(t) = \frac{1}{2}E_{\max}\langle\psi_0|\mathbf{e}\cdot\hat{\boldsymbol{\mu}}_{\mathrm{tot}}|\psi_i\rangle\, e^{\frac{-t^2}{2\sigma_t^2}}\,[e^{i\omega_{\mathrm{c}}t}+e^{-i\omega_{\mathrm{c}}t}]. \tag{5}$$

Through treating the pulse as a time-dependent perturbation to the first order that decays to zero sufficiently fast with time, it can be shown that the expansion coefficient of a single excited eigenstate after the pulse is [6]:

$$c_i(t=\infty) = -\frac{i}{\hbar}\int dt e^{i\omega_{i\leftarrow 0}t}V_{i\leftarrow 0}(t) \tag{6}$$

where $\omega_{i\leftarrow 0}$ is the angular frequency of the $i \leftarrow 0$ electronic transition. By substituting Eq. 5 into Eq. 6 and evaluating the resulting Fourier transforms, we obtain the following expression for the expansion coefficient of eigenstate i:

$$c_i(t=\infty) = \frac{\sqrt{\pi}E_{\max}}{\sqrt{2}\hbar\sigma_\omega}\langle\psi_0|\mathbf{e}\cdot\hat{\boldsymbol{\mu}}_{\mathrm{tot}}|\psi_i\rangle\,[e^{\frac{(\omega_{i\leftarrow 0}-\omega_{\mathrm{c}})^2}{2\sigma_\omega^2}}+e^{-\frac{(-\omega_{i\leftarrow 0}-\omega_{\mathrm{c}})^2}{2\sigma_\omega^2}}] \tag{7}$$

where the two Gaussian distributions of the angular frequencies $\omega_{i\leftarrow 0}$ and ω_{c} are derived from the Fourier transform of the time-dependent coupling term, $V_{i\leftarrow 0}(t)$, in Eq. 6, where the phase factor is given by $e^{i(\pm\omega_{i\leftarrow 0}-\omega_{\mathrm{c}})t}$. The latter term in the square brackets of Eq. 7 is typically neglected through the rotating wave approximation, which asserts that high frequency terms integrate to (almost) zero. In this case, the latter term corresponds to a high angular frequency, as $\omega_{i\leftarrow 0} > 0$ in photon absorption. By squaring Eq. 7, we obtain the relative probability of the pulse accessing eigenstate i from the ground state at a fixed nuclear configuration ($\mathbf{R}$) [6]:

$$P_{\mathbf{R}}(i) = |c_{\mathrm{i}}(t=\infty)|^2 = \frac{\pi E_{\max}^2}{2\hbar^2\sigma_\omega^2}|\langle\psi_0|\mathbf{e}\cdot\hat{\boldsymbol{\mu}}_{\mathrm{tot}}|\psi_i\rangle|^2 e^{\frac{(\omega_{i\leftarrow 0}-\omega_{\mathrm{c}})^2}{\sigma_\omega^2}} \tag{8}$$

To estimate the transition dipole moment associated with the $i \leftarrow 0$ transition, we express the excited eigenstate i in the diabatic basis, $|\psi_i\rangle = \sum_{k=1}^{M_{\mathrm{A}}} U_{ki}^{\mathrm{XT}}|\phi_k^{\mathrm{XT}}\rangle + \sum_{m=1}^{M_{\mathrm{D}}}\sum_{n=1}^{M_{\mathrm{A}}} U_{(mn)i}^{\mathrm{CT}}|\phi_{mn}^{\mathrm{CT}}\rangle$, where the expansion coefficients U_{ki}^{XT} and $U_{(mn)i}^{\mathrm{CT}}$ are the elements of the unitary matrix transforming from the adiabatic to the diabatic states. This gives for the transition dipole moment:

$$\langle\psi_0|\mathbf{e}\cdot\hat{\boldsymbol{\mu}}_{\mathrm{tot}}|\psi_i\rangle = \langle\psi_0|\mathbf{e}\cdot\hat{\boldsymbol{\mu}}_{\mathrm{tot}}|\sum_{k=1}^{M_{\mathrm{A}}} U_{ki}^{\mathrm{XT}}|\phi_k^{\mathrm{XT}}\rangle + \sum_{m=1}^{\mathrm{M_D}}\sum_{n=1}^{\mathrm{M_A}} U_{(mn)i}^{\mathrm{CT}}|\phi_{mn}^{\mathrm{CT}}\rangle\rangle \tag{9}$$

$$\approx \langle\psi_0|\mathbf{e}\cdot\hat{\boldsymbol{\mu}}_{\mathrm{tot}}|\sum_{k=1}^{M_{\mathrm{A}}} U_{ki}^{\mathrm{XT}}|\phi_k^{\mathrm{XT}}\rangle\rangle \tag{10}$$

$$\approx \sum_{k=1}^{M_{\mathrm{A}}} U_{ki}^{\mathrm{XT}}\mathbf{e}\cdot\boldsymbol{\mu}_k \tag{11}$$

$$\approx \sum_{k=1}^{M_{\mathrm{A}}} U_{ki}^{\mathrm{XT}}\mathbf{e}\cdot\sum_{K\in k} q_K^{\mathrm{T}}\mathbf{R}_K. \tag{12}$$

In Eq. 10 the small contribution of CT states is neglected, in Eq. 11 the total transition dipole is approximated by a sum of molecular transition dipoles for the S1 $\leftarrow$ S0 transition of PDI molecules and in Eq. 12 the molecular transition dipole is approximated by the atomic transition charges (q_K^{T}), similarly to the calculation of excitonic couplings, Eq. 12 main text. A similar approach for the calculation of the transition dipole moment was taken in Ref. [7]. The exponential term in Eq. 8 denotes the probability of a photon with the requisite frequency for the $i \leftarrow 0$ transition, which can be calculated with the knowledge of the energy of the excited eigenstate relative to the ground state.

Simulation details. To obtain $P(\mathbf{R})$ we sampled 1000 geometries (every ps) from a 1 ns classical NVE run of the neutral ground state of the a6T:PDI interface at 300 K, where we used the GAFF [8] force-field for neutral a6T and PDI molecules. In regard with the calculations of $P_{\mathbf{R}}(i)$, we note that Eq. 8 cannot be directly applied to the electronic Hamiltonian calculated in X-SH because according to Eqs. 9 and 11 in the main text the site energies of diabatic states are expressed relative to the interfacial CT-state, not the ground state. Therefore, we calculate the relative energy of the excited eigenstates of $\hat{H}(\mathbf{R})$ by shifting the diagonal elements of $\hat{H}(\mathbf{R})$ such that the exciton site energies will equal the S1 $\leftarrow$ S0 transition energy of a single gas-phase PDI molecule in its lowest energy neutral configuration ($\mathbf{R}_{\mathrm{GS}}$). We approximate this with the S1 $\leftarrow$ S0 transition energy (specifically the 0-0 vibrational transition) of a PDI derivative in toluene solution, which is 2.36 eV according to Ref. [9].

This means that the site energy of the exciton corresponds to the S1 energy relative to S0 plus the energy for displacing the nuclei from $\mathbf{R}_{\mathrm{GS}}$ to $\mathbf{R}$. This holds for the CT site energies as well, as their lower electronic energy (relative to the exciton) is already incorporated into the expression for the site energy of the exciton before we apply this linear shift. We calculated the (site-energy shifted) electronic Hamiltonian of each sampled nuclear configuration, and then diagonalised it to obtain its resulting eigenstates. Our electronically active region is a 1-dimensional chain of 20 a6T and 20 PDI molecules, which results in 420 localised quasi-diabatic states constituting the electronic state space. Therefore, sampling 1000 geometries results in an ensemble of 420,000 possible excited eigenstates. We then calculated the transition dipole moment associated with each eigenstate by passing the atomic coordinates of the PDI molecules into Eq. 12. To visualise the distribution of the transition dipole moments of the eigenstates, hereafter referred to as μ_{Tr}, we sorted the eigenstates in the ensemble into 200 energy bins and then calculated the average μ_{Tr} of each energy bin. In Fig. 1a, we have plotted the average μ_{Tr} as a function of eigenstate energy (relative to the neutral ground state in $\mathbf{R}_{\mathrm{GS}}$) for the electronic Hamiltonian of simulation (1) in Table 2 of the main text. As shown in Fig. 1a, we have chosen the central wavelength of the pulse such that it maximally overlaps with the peak of μ_{Tr}. We have calculated the pulse width by following the procedure outlined in Ref.[10], where the uncertainty associated with the temporal pulse length and frequency width is given by $\Delta\omega\Delta t = 1/2$. We have set $\Delta t = 10$ fs, which is a pulse length that can be achieved using commercial spectroscopy equipment. The same strategy was used to determine the initial conditions for all other simulations (2)-(7) in Table 2 in the main text. After obtaining the ensemble of possible eigenstates, we calculated the

relative excitation probability of each eigenstate by inserting its transition frequency and transition dipole moment into Eq. 8 and re-normalising the probability obtained from each eigenstate. The initial eigenstates (and underlying nuclear configurations) of the 500 X-SH trajectories were then randomly sampled from the ensemble, with the sampling weighted by the eigenstates' excitation probabilities. The histogram of the energies of initial eigenstates selected for the unmodified heterojunction (simulation (1) in Table 2 main text) is shown in Fig. 1b, and is overlayed with the average excitation probability of 200 energy bins of possible eigenstates. As expected, the energy distribution of eigenstates, which were selected with the procedure outlined above, follows the excitation probability as a function of eigenstate energy. Fig. 1c breaks down the total μ_{Tr} shown in panel a into the contributions of eigenstates categorised as XT, CT or hybrid XT-CT, where each state contribution has been independently renormalised to render the curves comparable. The eigenstates have been categorised with the procedure outlined in the main text. The similarity in shape between the XT contribution and total μ_{Tr} indicates that XT eigenstates typically have the highest transition dipole moment, with the peak in μ_{Tr} coinciding with the upper edge of the XT band (Main Fig. 2a). The peak of the CT states' contribution to the total μ_{Tr} coincides with the energies of the iCT states, as these are most likely to hybridise with XT states in simulation (1), as shown by Main Fig. 3.

Supplementary Discussion 3: Exciton binding energy

The exciton binding energy (E_{b}), as defined in Eq. 24 of the main text, is a key parameter that illuminates the relative energetics of XT and CS states in a given system. Each term in the expression for E_{b} is usually defined with respect the neutral ground state, but can also be defined with respect to any other energetic baseline. In this case, we express the optical gap ($E_{\text{g}}^{\text{opt}}$) and the transport gap (E_{f}) with respect to the iCT state at $\mathbf{R}_{\text{GS}}$. The energy of the uncorrelated electron hole pair with respect to the iCT state at $\mathbf{R}_{\text{GS}}$ is equal to the limit of the Coulomb interaction (with no field) at infinite distance, as there are no thermal fluctuations affecting the relative energies of different CT states. $E_{\text{g}}^{\text{opt}}$ can be estimated by taking the lowest eigenstate energy when the total eigenstate excitation probability (plotted in Fig. 1b) is non-zero. To express this eigenstate energy with respect to the iCT state at $\mathbf{R}_{\text{GS}}$, we subtract the eigenstate energy by the iCT energy at $\mathbf{R}_{\text{GS}}$, which is given as the S1 excitation energy of a PDI molecule (2.36 eV) subtracted by the XT-CT energetic offset (defined as ϵ^{XT} in the main text) at $\mathbf{R}_{\text{GS}}$. Therefore, after expressing each term with respect to the iCT state, we insert them into Eq. 24 of the main text to obtain an estimate for E_{b}. For simulation (1), $E_{\text{opt}} = 565$ meV; this value will remain similar for a given value of ϵ_{r} and different electronic couplings between CT states, since the peak of the total μ_{Tr} is mainly controlled by the transition dipole moments of the excitons (Figs. 1a and 1c). This results in simulations with equal ϵ_{r} and different electronic couplings displaying small deviations in E_{b}.

Supplementary Discussion 4: Exciton decay and charge generation for simulations (1)-(7) in Table 2 main text

Main Fig. 4 displayed the time-dependent contributions of 4 different diabatic state-types (given by P_{XT}, P_{iCT}, P_{niCT}, and P_{CS}, Eqs. 17-20, main text) to the electronic wavefunction $\Psi(t)$, for simulations (1), (2) and (5) in Table 2. This showed that increasing the electronic coupling between CT states or reducing E_{b} results in a higher rate of exciton dissociation and charge generation (such that it can be resolved on a 10 ps timescale). Here, Fig. 2 shows such electronic populations for simulations (1) - (7), overlaid with the exciton decay profile obtained through the least-squares fitting of a bi-exponential decay function (Eq. 1, main text) to P_{XT}. The electronic populations of simulations with unmodified electronic couplings and a progressively weaker Coulomb electron-hole interaction (simulations (1,4,7)) correspond to panels a-c, which reaffirms that a weaker Coulomb barrier results in faster exciton decay and charge generation, alongside a general increase in the non-interfacial character of CT states following exciton dissociation. The increase in exciton dissociation rate is quantified by panels f and i, which display the fitted decay constants and relative contributions, respectively, for bulk (b) and interfacial (i) exciton dissociation types, as delineated in Eq. 1 of the main text. A reduction in E_{b} from 260 to -310 meV accelerates both dissociation types, with k_{b} and k_{i} doubling and tripling, respectively, whilst their contributions remain approximately constant.

Panels d and g (simulations (2) and (3)) correspond to simulations with E_b = 260 meV and progressively increased electronic couplings. Furthermore, panels e and h (simulations (5) and (6)) display the electronic populations when E_b = 10 meV and the coupling is also strengthened. Both sets of parameter regimes indicate faster exciton dissociation and charge generation as a result of stronger electronic coupling between CT states, with P_CS actually overtaking P_niCT and P_iCT formation when an adequate balance between electronic coupling and E_b is achieved (panels e and h). In all cases where P_CS becomes significant, P_iCT remains comparable, since the Coulomb interaction continues to push niCT states higher in energy. As in the case of increasing E_b without modifying the coupling, increasing the electronic coupling strength at a given E_b results in a comparable increase in k_b and k_i, with panel i also suggesting that a higher proportion of bulk excitons are initially excited as the CT coupling is increased.

Supplementary Discussion 5: Least-squares Fitting

The least-squares fitting of the electronic exciton populations P_XT was undertaken with the *least_squares* function of the *scipy.optimize* [11] python module. We used an exponential decay function to compute the residuals, as we assumed that the rate of exciton decay is linearly proportional to the proportion of trajectories that are in an excitonic state at any given time. The initial guesses of the decay constants and coefficients of each exponential decay term were found through preliminary visual fitting, before being passed into the algorithm. We have used the Trust-region Reflective Algorithm [12] available within the *scipy.optimize* module with no bounds on the decay constants. We used a linear loss function, whose tolerance was set to 10^{-8}. The use of a bi-exponential decay function corresponds to the assumed presence of two non-overlapping subsets of excitons in each swarm of trajectories, which are characterised by two (potentially) different decay constants. The use of a tri-exponential decay function similarly corresponds to 3 independent subsets of excitons. To validate our use of a bi-exponential decay function, we attempted to fit the exciton population of each simulation to an exponential decay function of 1,2 and 3 decay terms, and compared their ability to account for the underlying data with their R^2 values. The chosen bi-exponential decay function was outlined in Eq. 1 of the main text. However, in the fitting function passed into the fitting algorithm, the ratio(s) of coefficients was used as a fitting parameter instead of the coefficients themselves. For the decay functions with more than 1 term, we used the ratio(s) to calculate the expansion coefficients such that their sum is always equal to P_XT at $t = 0$.

Fig. 3a displays the R^2 values obtained through the application of all three aforementioned decay functions. Increasing the number of terms in the decay function results in a general increase in R^2, but this becomes negligible once more than 2 exponential decay terms are included. Fig. 3b plots the decay constants associated with the mono-exponential and bi-exponential fits, and suggests that the increased R^2 value is due to the inclusion of a small subset of excitons that dissociate rapidly, likely because they are excited near the heterojunction interface and are therefore not limited by exciton diffusion. Therefore, the two subsets of excitons represented by the

bi-exponential fit are labelled by the subscripts i (interfacial) and b (bulk), as written in Eq. 1 of the main text.

Panels c and d show the decay constants obtained by fitting the tri-exponential decay function to P_{XT} for each parameter regime. In addition to the R^2 value, the behaviour of exciton dissociation is also broadly unaffected by the inclusion of a third decay term in the fitting process. All sets of decay constants in panel c are still similarly split into fast and slow components, with virtually no change in the constants corresponding to bulk exciton dissociation. Furthermore, the contribution of exciton dissociation from the bulk remains dominant for each parameter regime.

Supplementary Discussion 6: Exciton Dissociation Distance

Main Fig. 5 depicts a new secondary mechanism by which an exciton can dissociate if the coupling between CT states is large enough. This involves the exciton directly transitioning to transiently delocalised hybrid XT-CT states, which are converted into niCT and then CS states in what we term hot exciton dissociation. To establish the statistical significance of such a pathway, we evaluate the expectation value of the exciton's displacement with respect to the interface for each X-SH trajectory, at the timestep immediately preceding the onset of exciton dissociation. For a given X-SH trajectory, we track P_{XT} (Eq. 17, main text) throughout the 10 ps dynamics and obtain the timestep before which P_{XT} decreases below 0.95, given that P_{XT} then subsequently decreases below 0.05 and then stays below 0.5 for the next 500 fs. For a single trajectory, the time-dependent expectation value of the exciton distance (with respect to the interface) is defined as

$$r_{\mathrm{XT}}^{\mathrm{rel}}(t) \approx \langle \Psi(t) | \hat{x}^{\mathrm{rel}} | \Psi(t) \rangle \approx \frac{\sum_{k=1}^{\mathrm{M_{XT}}} |u_k^{\mathrm{XT}}(t)|^2 (x_{k,0}^{\mathrm{PDI}} - x_{\mathrm{int}})}{\sum_{k=1}^{\mathrm{M_{XT}}} |u_k^{\mathrm{XT}}(t)|^2} \tag{13}$$

where $\hat{x}^{\mathrm{rel}}$ is the operator for the x-coordinate relative to the midpoint between the interfacial a6T and PDI molecules, x_{int}. Since the electronically active region mainly extends across a single crystallographic (a) direction, the total Euclidean distance between the exciton centre and interface ($r_{\mathrm{XT}}^{\mathrm{rel}}(t)$) is well approximated by the distance along this direction, which is given here by the x-axis. The expectation value of this distance is equivalent to the sum of individual PDI molecules' centre of mass x-coordinates with respect to the x-coordinate of the interfacial a6T-PDI midpoint (given by $x_{k,0}^{\mathrm{PDI}} - x_{\mathrm{int}}$), weighted by the squared coefficients of the corresponding quasi-diabatic XT states. Each coefficient has been renormalised to remove the impact non-zero CT state amplitudes may have on $r_{\mathrm{XT}}^{\mathrm{rel}}(t)$. We approximate the x-coordinate of each PDI molecule at time t as its initial coordinate at $t = 0$, $x_{k,0}^{\mathrm{PDI}}$, since the geometric centres of the upper / lower rings of each molecule are constrained throughout the dynamics. Fig. 4 therefore displays the distributions of exciton distances upon dissociation for each simulation in the main text. The exciton distances are divided into 3 bins: 5-35Å, 35-65Å, and 65-95Å. The height of each bar denotes the number of trajectories with excitons dissociating within the respective distance bin, whilst the coloured section of each bar denotes the proportion of excitons in this bin that have relaxed into

a given final state after 10 ps. Panels a-c show the exciton distance distributions as the Coulomb interaction is progressively weakened without the coupling between CT states being modified (simulations (1,4,7)). As the Coulomb interaction weakens, both hot and cold exciton dissociation pathways are resolved within 10 ps, with an increasing proportion of excitons relaxing into higher-energy niCT states, which are either accessed directly from the exciton or after dissociation into an iCT state. Crucially, the notably faster exciton decay at $E_\mathrm{b} = -310$ meV compared to 260 and 10 meV, is due to $\approx 30\%$ of excitons now dissociating more than 3.5 nm away from the interface, thereby mitigating the rate-limiting nature of exciton diffusion. Panels d and f correspond to the exciton distance distributions when$E_\mathrm{b} = 260$ meV and the electronic coupling is doubled and tripled, respectively (simulations (2) and (3)). Panels e, and g display distributions with the same coupling pattern with $E_\mathrm{b} = 10$ meV (simulations (5) and (6)). In both cases, an increase in electronic coupling strength results in increasingly non-interfacial exciton dissociation and hot exciton dissociation, with a reduction in E_b continuing to facilitate both phenomena at higher couplings. In each simulation, excitons dissociating further from the interface are more likely to access the CS state after 10 ps. This may occur because non-interfacial XT states are relatively more likely to transition to delocalised hybrid XT-CT states with non-interfacial XT (and therefore niCT, see Fig. 8) character, compared to XT states centred closer to the interface, which are more likely to strongly couple to CT states also centred around the interface. The non-interfacial CT character of such delocalised hybrid states renders them more likely to directly transition to niCT and then CS states, completing the hot dissociation pathway.

Supplementary Discussion 7: Exciton Relaxation Pathways

Main Fig. 5 indicates that, upon increasing the electronic coupling between CT states (or reducing E_b), a new common trajectory emerges that displays non-interfacial hot exciton dissociation. This is further indicated by the average active eigenstate increasing in energy following exciton dissociation, as well as the electronic niCT population increasing (Main Fig. 4). Additionally, the pathways taken by each trajectory as the exciton relaxes can be assessed by tracking $\Psi(t)$ each timestep over the course of the X-SH dynamics. Each timestep, $\Psi(t)$ is categorised as one of the 5 states used in the main text to classify the eigenstates of the electronic Hamiltonian (XT, iCT, niCT, CS, hybrid XT-CT). Then the identity of $\Psi(t)$ is known for each timestep of a trajectory, and the relaxation pathway the exciton in a trajectory takes is determined by the identity of $\Psi(t)$ at 10 ps and the electronic states occupied during the rest of the trajectory. We categorise the evolution of $\Psi(t)$ into 6 general pathways, where Fig. 5 shows the percentage of (dissociated) trajectories accessing each pathway, for different electronic couplings and values of E_b. Panel a indicates that, as E_b is reduced, both hot and cold exciton dissociation pathways (pathways 6 and 5 in Fig. 5, respectively) occur on the 10 ps timescale and a larger proportion of excitons are dissociating directly into niCT states. Panels b and c reiterate this trend when the electronic coupling is increased at $E_\mathrm{b} = 260$ meV or $E_\mathrm{b} = 10$ meV, respectively. The hot pathway

begins to dominate over the cold pathway and increasingly fewer excitons are accessing niCT states via the iCT state first; this is due to the fact that increasing the CT coupling magnitude delocalises the charge carriers to a larger extent compared to an isolated reduction inE_b. However, an important point to consider is that $\Psi(t)$ is classified as an iCT state only if $P_\mathrm{iCT} > 0.8$ at time t. As the charge carrier delocalisation increases, this criterion is less likely to be satisfied even if the electron-hole pair is located close to the interface (such that P_iCT is still close to 0.8). This will therefore result in the contribution of the cold pathway being slightly underestimated as the electronic coupling is increased. However, the bulk of excitons dissociating via the hot pathway likely proceed via niCT states centred nanometres away from the interface, since the average electron-hole distance of the hybrid states' CT components increases by over 2 nm as the electronic coupling is increased (Fig. 7b).

Supplementary Discussion 8: Characterisation of Hybrid XT-CT State

Main Fig. 3 (and Fig. 5) showed that the hybrid XT-CT state is an essential intermediate between pure XT and CT states. Increasing the electronic coupling between CT states or reducing E_b further demonstrated that, as more delocalised hybrid states become thermally accessible, they become the gateway to the hot, non-interfacial exciton dissociation. We therefore examine more closely how changes in the aforementioned parameters can affect the extent of delocalisation and non-interfacial character of hybrid XT-CT states.

As the hybridisation of $\Psi(t)$ is due to the occupancy of an active eigenstate with hybrid XT-CT character, we construct a representative distribution of hybrid eigenstates in each simulation. We sample the eigenstates of $\hat{H}(\mathbf{R})$ of each trajectory every 10 timesteps, and only select the eigenstates that satisfy the criterion for a hybrid XT-CT state, as outlined in the main text. For each simulation, the resulting ensemble of hybrid eigenstates was then organised into bins of electron-hole distance of the CT component, which range from 0 to 12 nm in increments of 2 nm. The electron-hole distance of a given state was calculated with Eq. 13 but replacing the Euclidean distance between the exciton and the interface with the Euclidean distance between the electron and hole, and renormalising with respect to CT populations. The IPR of each hybrid XT-CT state was calculated with Eqs. 21-23 in the main text, with the mean IPR of all states within a given distance bin being calculated subsequently. The mean exciton IPR values of the hybrid eigenstates, whose electron-hole distances fall into a given bin, are plotted in Fig. 6a, where the x-coordinate of each point corresponds to the midpoint of a distance bin. The electron-hole distance bins were truncated to 12 nm or 8 nm, depending on the simulation, despite the maximum possible electron-hole distance being just under 2 nm. This is due to the number of hybrid XT-CT states with electron-hole distances above these 2 cutoffs being insufficient for a converged IPR. Figs. 6b and 6c show the same plot, but for electron and hole IPR, respectively.

Fig. 6 indicates that the XT and CT contributions to hybrid XT-CT states become more delocalised as the CT contribution becomes centred away from the interface. This suggests that increased hybrid state delocalisation is more likely to result in direct

transitions to niCT states, and then CS states. We notice that the hole IPR (Fig. 6c) begins to decrease once the electron-hole distance is sufficiently large. This is likely due to the hole, which is delocalised on ≈ 3 more molecules than the electron at this distance, being constrained by the heterojunction boundary once it is sufficiently far from the interface.

Although more delocalised hybrid XT-CT states are more likely to directly transition into niCT states (per Fig. 6), such eigenstates must then become more abundant or thermally accessible as the electronic coupling is increased, to explain the hybrid states' role in increasing the CS yield (see Table 2, main text). Fig. 7a displays the average exciton IPR calculated from the full hybrid state distribution sampled from X-SH runs (1) - (7). The exciton IPR indeed increases alongside the electronic coupling (or reduction in E_b), which suggests that the average hybrid XT-CT state does exhibit more delocalisation and is hence more likely to transition to niCT states and undergo hot exciton dissociation. Similarly, the average electron-hole distances obtained from the full hybrid state distributions (Fig. 7b) depict the same trend, consistent with relationship between IPR and electron-hole distance illustrated in Fig. 6.

It was shown in Fig. 4 that excitons dissociating away from the interface are more likely to undergo the hot dissociation pathway. Main Fig. 5 also indicates that the XT component of the transient hybrid state has significant amplitude away from the interface. These two factors suggest that hybrid states with non-interfacial XT components are also more likely to have non-interfacial CT components. We therefore calculate the exciton distance (with respect to the interface) of each sampled hybrid XT-CT eigenstate with Eq. 13 and paritition the ensemble of hybrid states into 3 distance bins: 5-35Å, 35-65Å, and 65-95Å. For the subset of hybrid states within each distance bin, we then calculate the average electron-hole distance. We carry out this procedure for simulations with a significant CS yield, namely (3) and (5) - (7), the result of which is plotted in Fig. 8. Indeed, the average electron-hole distance (of the hybrid state CT components) increases alongside the exciton distance, which suggests that hybrid states with more non-interfacial XT contributions are more likely to directly transition to niCT states, and then CS states.

Supplementary Discussion 9: Analysis of Non-adiabatic Couplings involving Hybrid XT-CT States

In addition to the density of thermally accessible states, the probability of a transition from an active eigenstate to an niCT state is linearly proportional to the non-adiabatic coupling element (NACE) between the two states. The non-adiabatic coupling refers to the electronic coupling between two eigenstates of the electronic Hamiltonian at a given nuclear geometry, as the thermal motion of the nuclei (with time) changes the electronic structure of the system and its resultant eigenstates. This is distinct from the aforementioned electronic coupling, which we have used to refer to the off-diagonal matrix elements between localised electronic states of the electronic Hamiltonian in the quasi-diabatic basis. The NACE between an active eigenstate, labelled a, and state j is defined as $d_{ja}^{\mathrm{adiab}} = \langle\psi_a| \frac{\partial}{\partial t} |\psi_j\rangle$. The NACE is also inversely proportional to ΔE_{ja},

which is the potential energy difference between states a and j [13]. The NACE is proportional to the overlap between the active eigenstate and the time-derivative of another state j, which becomes non-zero if state j evolves such that there is a common region of excitation density between the two states - this was shown to be crucial in the transitions from non-interfacial XT to hybrid XT-CT states. Hybrid XT-CT states with more niCT character are more likely to facilitate non-interfacial exciton dissociation, and transition into pure niCT states, which are the precursors to CS states. We therefore focus on the NACEs between XT and hybrid XT-CT states, and between hybrid XT-CT states and pure CT states (iCT, niCT and CS). Due to the NACE being inversely proportional to ΔE_{ja}, we anticipate that it should generally decrease as hybrid or CT states are centred further from the interface. To account for the joint effect of higher NACEs and density of accessible states, we calculate the sum of Boltzmann-weighted NACEs between a given initial and final state-type, averaged over all trajectories and timesteps of each simulation in this work. The sum of Boltzmann-weighted NACEs between a given currently occupied state, a, and a subset, b, of possible future states, for a single timestep, is defined as:

$$D_{ba} = \sum_{j \in b} d_{aj}^{\mathrm{adiab}} e^{\frac{-\Delta E_{ja}}{k_{\mathrm{B}}T}} \tag{14}$$

where j refers to a state within subset b, and the exponential term in each element of the NACE sum D_{ba} is the thermal weighting applied to each NACE term within the subset of possible future states.

In Fig. 9a, we plot the thermally averaged D_{ba} for the transition between an initial XT state, and different sets of hybrid XT-CT states resolved by their expectation value of the electron-hole distance, as calculated in Supplementary Discussion 8. As expected, D_{ba} decreases alongside electron-hole distance for all parameter regimes, due to the higher energy of niCT states. Crucially, the gradient of descent is significantly reduced as the electronic coupling is increased or E_{b} reduced. For example, the difference in D_{ba} between simulations (1) and (5), where (5) exhibits a lower E_{b} and stronger electronic coupling than (1), reaches an order of magnitude when the electron-hole distance of the hybrid XT-CT state exceeds 2 nm. The minimum distance bin, corresponding to hybrid XT-CT states with electron-hole distances between 1 nm and 2 nm, remains with the highest weighted NACE sum, which may contradict the relative dominance of niCT and CS states observed in the population dynamics of simulation (5). However, this is due to the bin in question still containing hybrid states with niCT character, as the interfacial electron-hole distance is only 0.98 nm. Fig. 9b plots D_{ba} between an initial active hybrid XT-CT eigenstate, and sets of pure CT states resolved by their electron-hole distance. Regarding simulation (1), all NACE sums are similarly small, as every distance bin contains niCT states which are thermally inaccessible due to the strength of the Coulomb interaction. The smallest distance bin also has a small D_{ba} because it likely undersamples the iCT state, to which virtually all hybrid states relax. This is because the direct electronic coupling between the interfacial quasi-diabatic XT state and iCT state can result in a hybrid XT-CT state of mostly iCT character, which subsumes the pure iCT state in the electronic structure of the junction during the time frame of exciton dissociation. The XT character of

such a hybrid state is subsequently reduced by the reorganisation of the a6T and PDI molecules, which effaces the hybridisation caused by the electronic coupling between diabatic states. As observed for the D_{ba} for exciton-to-hybrid transitions in panel a, the D_{ba} values of hybrid-to-CT transitions increase for all electron-hole distances as the electronic coupling is increased or E_{b} decreased. This indicates that niCT states are indeed more likely to be accessed directly from exciton dissociation, from which the CS can be accessed via the hot pathway.

Supplementary Discussion 10: Convergence of Exciton Dissociation with Respect to Number of Trajectories

Each simulation type was carried out with 500 independent trajectories. Each trajectory was propagated for 10 ps, to sample as much of the exciton relaxation process as possible, under the current computational constraints. A key result obtained from our study is the advent of non-interfacial exciton dissociation as a function of the energy landscape of the CT-band. The electronic structure of X-SH is embedded within the Fewest-switches Surface Hopping [14] framework, where each trajectory evolves stochastically based on the instantaneous transition probability from the current active state to any other state that is sufficiently close in energy. Due to the stochasticity of the trajectories, events (such as non-interfacial exciton dissociation) that do not reflect the broad physics of the system may still be observed, but their contribution to thermal properties should be effaced through adequate sampling of the phase space. Therefore, it is paramount to ensure that the manner of exciton dissociation is converged with respect to the number of trajectories in each simulation. This can be checked by plotting the accumulated average of the exciton dissociation distance, as well as the accumulated standard error, as a function of the number of trajectories. The exciton dissociation distance of each trajectory is calculated by first obtaining the final timestep before the total electronic wavefunction evolves into a hybrid state from a pure exciton state, in the process of its full conversion into a CT-state, which persists for at least half a picosecond afterward. At this timestep, the expectation value of the exciton's distance (with respect to the interface) is calculated, by calculating the sum of each PDI molecule's x-coordinate weighted by the squared expansion coefficient of the associated exciton diabat. Fig. 8 displays the accumulated averages (lines and dots) and standard errors (shaded region) of the exciton dissociation distance for simulations (1) - (7), from which it is apparent that convergence has been achieved for all simulation types. For simulations where most excitons are dissociating near the interface, such as (1), the standard error remains small and effectively constant throughout the averaging process, as all trajectories are effectively dissociating next to the interface. Conversely, the standard error is initially much larger for simulations exhibiting non-interfacial dissociation, such as (6) and (7), as the range of possible dissociation distances now spans the entire PDI phase.

The relative contributions of the hot and cold charge generation pathways to each simulation type are obtained by tracking the evolution of the electronic wavefunction $\Psi(t)$ of each trajectory, and categorising the decay pathway of the trajectory by the series of electronic state-types it occupies over the time frame of the dynamics. It was

also observed that the incidence of hot exciton dissociation significantly increases as the density of niCT states increases at the energy level of the exciton.

To ensure that this trend is also representative of the underlying dynamics, we have plotted the percentage contributions of the hot and cold charge generation pathways (with respect to all dissociated trajectories) as a function of the total number of trajectories in Figs. 11a and 11b, respectively. The percentage of trajectories proceeding via the cold pathway converges after 400 trajectories, whilst the percentage of trajectories proceeding via the hot pathway converges after 300. We have plotted the convergence for simulations (2) - (7), as simulation (1) does not exhibit charge separation on the 10 ps timescale due to the strength of the Coulombic electron-hole interaction. It should be noted that the contributions of the different mechanisms are specifically converged with respect to the simulation time of 10 ps, as the majority of trajectories are still in iCT or niCT states by the end of each simulation.

Supplementary Discussion 11: Convergence of Exciton Dissociation with Respect to Nuclear Timestep

The nuclear positions in X-SH are propagated with the velocity Verlet algorithm. X-SH is always propagated in the NVE regime, so the total system energy should be conserved throughout the dynamics. The contributions of the nuclear gradients of the electronic CT and XT-CT couplings with respect to the total atomic forces constitute the largest source of energy drift in X-SH, as these are calculated via finite differences at each nuclear timestep. Conversely, the nuclear gradients of the diagonal elements of the electronic Hamiltonian and the excitonic couplings can be calculated analytically, such that their contribution to the energy drift will be orders of magnitude smaller. Therefore, one must carefully tune the nuclear timestep of X-SH such that any further reductions in timestep return a miniscule improvement in energy conservation. We therefore performed a 300 fs X-SH run with a 2-dimensional active region in the a6T:PDI heterojunction, with 930 diabatic states (30 active donor/acceptor molecules). For 6 different nuclear timesteps ($0.01 - 0.13$ fs), we calculated the average energy drift of 100 trajectories by subtracting the final from the initial total energies, then dividing the difference by the number of atoms in the electronically active region and the simulation time in ps. Fig. 12 consistently stays below 10^{-7} E_h ps^{-1} atom^{-1}, which is similar to the energy drift values calculated in our previous work [15]. We therefore selected a nuclear timestep of 0.05 fs for all X-SH simulations included in the main text; this provides a good compromise between energy conservation and computational expense, as the compute time of X-SH scales inversely with the nuclear timestep.

To further ensure that the nuclear timestep is also small enough to detect potentially trivial crossings between eigenstates of the electronic Hamiltonian in FSSH, we repeated the first ps of X-SH dynamics with a nuclear timestep of 0.01 fs. This was undertaken for the simulations that displayed the lowest and highest CS yields, namely (1) and (6), as shown in Table 2 in the main text. To check that the manner of exciton dissociation was conserved upon the reduction of the timestep, Fig. 13a plots the

distribution of trajectories' electron-hole distances immediately following exciton dissociation, whilst 13b plots the corresponding distribution of exciton distances (with respect to the interface). At both timesteps, the vast majority of excitons still dissociate at the heterojunction interface, and form iCT states localised on the interfacial a6T:PDI pair. Fig. 13c displays the differences between electronic populations of the different types of states, as defined by Eqs. 17-20 in the main text, for 0.05 and 0.01 fs. A reduction of the timestep results in a minor reduction in the rate of exciton dissociation - and consequently a smaller P_{iCT} - but not enough to significantly change the main relaxation pathway of the exciton.

Simulation (6) is expected to display the highest density of CT states relative to the typical energy level of the relaxed exciton, and is therefore expected to be most affected by potential trivial crossings. Figs. 13d and 13e display the electron-hole and exciton distance distributions upon exciton dissociation. Both timesteps capture the increased probability of non-interfacial exciton dissociation and the direct population of niCT states thereafter, signified by the electron-hole distance distribution shifting to higher distances. The electronic population differences (Fig. 13f) indicate a slower reduction in P_{XT} upon going from a 0.05 to 0.01 fs timestep, and consequently smaller CT state populations. Despite this, the faster exciton dissociation and charge separation upon going from (1) to (6) is significantly larger than that predicted by Kinetic Monte Carlo simulations in the same parameter regimes. We therefore regard 0.05 fs as an efficient compromise between the effect of trivial crossings on the exciton dissociation mechanism and the sufficient sampling of statistics.

Supplementary Discussion 12: Validation of Electronic Coupling Calculation

As mentioned in the main text, the electronic coupling between CT states (via hole or electron transfer) or that between CT and XT states can be estimated through the approximate relationship $H_{ab} = \bar{C}\bar{S}_{ab}$, where $\bar{S}_{ab}$ refers to the overlap between the frontier orbitals involved in charge transfer (HOMO-HOMO or LUMO-LUMO overlap), and $\bar{C}$ is a constant of proportionality that can be scaled to maximise agreement between H_{ab} and $\bar{S}_{ab}$. The orbital overlap between a given pair molecules is rapidly calculated each timestep with AOM. We refer to previous work for a detailed explanation. To test the ability of AOM to capture the electronic charge-transfer coupling between a6T and PDI molecules, we sample 100 a6T and PDI dimers from a 1 ps X-SH run at 300 K. Fig. 14a plots the electronic coupling calculated for each dimer using Density Functional Theory (DFT) followed by Projection Operator-based Diagonalisation (POD), against the AOM-calculated overlap. The straight line plots $H_{ab} = \bar{C}\bar{S}_{ab}$ for the given overlap range and scaling constant used in AOM. Therefore, closer points to the line indicate a better agreement between POD and AOM. Good agreement is obtained between POD and AOM for CT electronic couplings via both HOMO-HOMO and LUMO-LUMO overlap.

We estimate the electronic coupling between the interfacial quasi-diabatic XT and iCT states, $H_{k,mn}^{\mathrm{XT-CT}}$, which facilitates hole transfer from the excitonic PDI to the neutral a6T molecule, by calculating the HOMO-HOMO overlap between the two

molecules with AOM. To test the ability of AOM in sufficiently capturing this coupling, we sample 25 interfacial a6T:PDI dimer geometries from the same 1 ps X-SH run, and calculate $H_{k,mn}^{\mathrm{XT-CT}}$ via TDDFT followed by MS-FED-FCD diabatisation. Upon plotting such couplings against AOM overlaps for the same dimer geometries, it is evident that AOM's performance has markedly decreased. However, since there is still a positive correlation between H_{ab} and $\bar{S}_{ab}$, we expect that AOM will broadly be able to capture the effect of thermal fluctuations on $H_{k,mn}^{\mathrm{XT-CT}}$, since the orbital interactions involved in hole transfer between the excitonic PDI and neutral a6T molecules are still pertinent to $H_{k,mn}^{\mathrm{XT-CT}}$.

Supplementary Discussion 13: Creation of Interfacial a6T:PDI Defect

The a6T:PDI heterojunction with the interfacial defect is shown in Fig. 15. The defect was manually created by taking the final geometry of the NVT equilibration run of the crystalline a6T:PDI heterojunction, deleting the second-most interfacial PDI molecule, and then rotating the interfacial PDI molecule until the ET and XT-CT couplings involving this molecule were reduced by a factor of 4 or more (compared to those reported in Table 1 in the main text). The XT coupling involving the interfacial PDI molecule was only reduced by $\approx 25\%$, since the Coulombic interaction between the S1 transition densities is less sensitive to distance than the orbital overlap. The restraints were then adjusted to reflect the new positions of the upper/lower rings of the interfacial PDI molecule (see *Methods* for information about the application of restraints). The adjusted heterojunction was then propagated in the neutral ground state for 1.1ns in the NVE regime, with the first 100 ps allowing the system to equilibrate (in case the intermolecular forces were significantly perturbed by the transformation of the molecule) whilst 1000 geometries were sampled each ps from the remaining 1ns of simulation time. The fluctuation of the temperature around 300 K was very similar to the NVE run of the fully ordered heterojunction.

Supplementary References

1. Spencer, J., Scalfi, L., Carof, A., Blumberger, J.: Confronting surface hopping molecular dynamics with marcus theory for a molecular donor–acceptor system. *Faraday Discuss.*, **195**, 215–236 (2016)

2. Giannini, S., Carof, A., Blumberger, J.: Crossover from hopping to band-like charge transport in an organic semiconductor model: Atomistic nonadiabatic molecular dynamics simulation. *J. Phys. Chem. Lett.*, **9**, 3116–3123 (2018)

3. Yang, H., Gajdos, F., Blumberger, J.: Intermolecular charge transfer parameters, electron–phonon couplings, and the validity of polaron hopping models in organic semiconducting crystals: Rubrene, pentacene, and C60. *J. Phys. Chem. C*, **121**, 7689–7696 (2017)

4. Blumberger, J.: Recent advances in the theory and molecular simulation of biological electron transfer reactions. *Chem. Rev.*, **115**, 11191–11238 (2015)

5. Kelley, A.M.: *Condensed-phase Molecular Spectroscopy and Photophysics.*, pp. 38–42. Wiley, Hoboken, N.J (2013)

6. Persico, M., Granucci, G.: *Photochemistry: A Modern Theoretical Perspective.*, pp. 87–100. Springer, Gewerbestrasse, Cham (2018)

7. Giannini, S. *et al.*: On the role of charge transfer excitations in non-fullerene acceptors for organic photovoltaics. *Mater. Today*, **80**, 308–326 (2024)

8. Case, D.A. *et al.*: AMBER 10. University of California (2008)

9. Bo, Y. *et al.*: Oxygen-Mediated Sequential Down-Conversion in Perylenediimides. *Artif. Photosynth.*, **1**, 226–236 (2025)

10. Donnelly, T.D., Grossman, C.: Ultrafast phenomena: A laboratory experiment for undergraduates. *Am. J. Phys.*, **66**(8), 677–685 (1998)

11. Virtanen, P. *et al.*: SciPy 1.0: Fundamental Algorithms for Scientific Computing in Python. *Nat. Methods*, **17**, 261–272 (2020)

12. Branch, M.A., Coleman, T.F., Li, Y.: A subspace, interior, and conjugate gradient method for large-scale bound-constrained minimization problems. *SIAM J. Sci. Comput.*, **21**, 1–23 (1999)

13. Baer, R.: Non-adiabatic couplings by time-dependent density functional theory. *Chem. Phys. Lett.*, **364**, 75–79 (2002)

14. Tully, J.: Molecular dynamics with electronic transitions. *J. Chem. Phys.*, **93**, 1061–1071 (1990)

15. Giannini, S., Ziogos, O.G., Carof, A., Ellis, M., Blumberger, J.: Flickering polarons extending over ten nanometres mediate charge transport in high-mobility organic crystals. *Adv. Theory Simul.*, **3**, 2000093 (2020)
